# Update on the Treatment of Gastric Cancer

**DOI:** 10.31662/jmaj.2018-0006

**Published:** 2018-09-28

**Authors:** Tadayoshi Hashimoto, Yukinori Kurokawa, Masaki Mori, Yuichiro Doki

**Affiliations:** 1 Department of Gastroenterological Surgery, Osaka University Graduate School of Medicine, Osaka, Japan

**Keywords:** gastric cancer, extended surgery, laparoscopic surgery, chemotherapy, molecular-targeted therapy, immunotherapy

## Abstract

Gastric cancer remains a major health concern worldwide, particularly in Asia. Surgery is the only curative treatment, and D2 gastrectomy is the standard therapy for resectable cases. Several clinical trials have been conducted in Japan to achieve higher cure rates via extended surgery; however, despite higher morbidity, none demonstrated prolonged survival. Against this background, minimally invasive surgical approaches that preserve gastric function and improve postoperative quality of life have been developed in recent years. For early gastric cancer, endoscopic resection and laparoscopic gastrectomy have achieved remarkable success even for later-stage cases. Long-term outcomes have been investigated in large-scale, randomized controlled trials. In addition, robot-assisted gastrectomy is now more common in clinical practice. S-1, an anti-tumor drug, is a key agent for treating gastric cancer and has resulted in dramatic improvements in survival. For locally advanced gastric cancer, patients are usually treated with surgery and adjuvant or neoadjuvant chemotherapy, and the efficacies of various regimens have been examined in many clinical trials. For unresectable or recurrent gastric cancer, new agents such as molecular-targeted agents and immune checkpoint inhibitors have emerged as notable treatments and are now being tested in numerous clinical trials. This review provides an update on gastric cancer treatment, highlighting current individualized strategies and future perspectives.

## Introduction

Gastric cancer is the fifth most common malignancy worldwide and the third leading cause of cancer-related deaths worldwide ^(1)^. While it is linked primarily to *Helicobacter pylori* infection, there are large regional differences in the incidence of gastric cancer; about 70% of cases occur in Asia, including Japan ^(2)^. Therefore, different gastric cancer treatments have been developed in Japan than in Western countries. Japanese surgeons have been influential in the development of gastric cancer surgery worldwide ^(3), (4)^, introducing techniques such as gastrectomy with extended lymphadenectomy. In the West, major treatment strategies include more limited lymph node dissection and chemotherapy or chemoradiotherapy ^(5), (6)^. Several clinical trials did not indicate more favorable outcomes following extended surgery in Japan ^(7), (8), (9), (10), (11)^; therefore, less invasive surgery has become preferable to minimize postoperative complications and preserve postoperative quality of life.

New chemotherapy regimens have been approved for gastric cancer, and new therapeutic strategies, such as molecular-targeted drugs and immune checkpoint inhibitors, are attracting great attention. In this review, we provide an update on gastric cancer treatment to illustrate current individualized strategies and future perspectives.

## Endoscopic Treatment

Endoscopic treatment, including endoscopic mucosal resection (EMR) and endoscopic submucosal dissection (ESD), is an alternative to surgery for early gastric cancer. Although there have been no phase III trials comparing the efficacy of endoscopic treatment with surgery, several studies have suggested comparable long-term outcomes ^(12), (13)^. In principle, endoscopic treatment is appropriate for gastric cancer cases wherein complete en-bloc resection is anticipated and there is almost no possibility of lymph node metastasis. The Japanese Gastric Cancer Association (4th edition) treatment guidelines state that the absolute indication criteria for EMR/ESD include patients with cT1a, without ulceration (UL0), and a tumor of ≤2-cm diameter histologically diagnosed as differentiated-type adenocarcinoma ^(14)^. More recently, because the JCOG0607 trial showed no post-ESD recurrence during long-term follow-up, the absolute indication criteria were expanded to include patients with any size cT1a differentiated-type adenocarcinoma without ulceration (UL0), and those with ≤3-cm cT1a differentiated-type adenocarcinoma with ulceration (UL1) ^(15)^. Moreover, the expanded indication criteria for ESD include patients with ≤2-cm cT1a undifferentiated-type adenocarcinoma and UL0; however, these should be considered preliminary criteria until favorable long-term outcomes after ESD are demonstrated in the ongoing JCOG1009/1010 trial ^(16)^.

## Extended Surgery

Investigators have studied the optimal surgical approach to gastric cancer in terms of the stomach resection extent and lymph node dissection range. Major phase III trials evaluating extended surgery for resectable gastric cancer are shown in Table 1. When systematic lymph node dissection was established in Japan in the 1960s, D2 lymph node dissection became standard during gastric surgery. However, the UK Medical Research Council found D2 lymph node dissection did not improve survival compared with D1 lymph node dissection; thus, lymphadenectomy of D1 or less has been standard in Western countries for many years^(17), (18), (19)^. However, in the long follow-up results of a Dutch trial, the recurrence rate of gastric cancer treated with D2 lymph node dissection was significantly lower than that treated with D1 lymph node dissection ^(20)^. In addition, a Taiwanese single-institute, phase III trial demonstrated a survival advantage of D2 compared with D1 ^(21)^. Therefore, D2 lymph node dissection is now recommended in the current National Comprehensive Cancer Network and European Society for Medical Oncology guidelines ^(22), (23)^.

**Table 1.** Major Phase III Trials of Extended Surgery for Resectable Gastric Cancer.

Trial name	Published year	No. of patients	Eligible patients	Treatment groups	Morbidity (%)	5-year OS (%)	P-value
MRC trial ^(17), (18)^	1996, 1999	737	cStage I-III	D1 D2	28 46	35 33	0.43
Dutch trial ^(19), (20)^	1995, 2010	1078	cStage I-III	D1 D2	25 43	45 47	0.53
Taiwanese trial ^(21)^	2006	335	cStage I-III	D1 D2	7.3 17.1	53.6 59.5	0.041
JCOG9501 ^(7)^	2008	523	cT3-T4b	D2 alone D2 + PAND	20.9 28.1	69.2 70.3	0.85
JCOG9502 ^(8), (9)^	2006, 2015	167	cT2-T4b (cardia/subcardia)	TH LTA	34.1 49.4	52.3 37.9	0.92^*^
JCOG0110 ^(10)^	2017	505	cT2-T4b (proximal)	Spleen-preservation Splenectomy	16.7 30.3	76.4 75.1	0.025^*^ (for non-inferiority)
JCOG1001 ^(11)^	2018	1204	cT3-T4a	Omentectomy alone Bursectomy	10.6 12.8	76.7 76.9	0.65^*^

* one-sided p-value. Abbreviations: OS, overall survival; HR, hazard ratio; PAND, para-aortic node dissection; TH, abdominal–transhiatal approach; LTA, left thoracoabdominal approach.

In Japan, several clinical trials have examined the superiority of extended radical surgery. The Phase III JCOG9501 trial evaluated the survival advantage of adding systematic para-aortic nodal dissection (PAND) to standard gastrectomy with D2 lymphadenectomy. The results showed that D2 lymphadenectomy plus PAND did not improve survival compared with D2 lymphadenectomy alone ^(7)^.

The Phase III JCOG9502 trial compared extended lower mediastinal node dissection, via the left thoracoabdominal approach (LTA), to limited dissection via the abdominal-transhiatal approach (TH) in patients with gastric cancer of the cardia or subcardia with esophageal invasion of ≤3 cm. However, LTA had no benefit in terms of survival, and there was higher morbidity than TH ^(8), (9)^.

The JCOG0110 trial evaluated the efficacy of splenectomy to dissect the splenic hilum nodes in proximal gastric cancer. Splenectomy did not improve survival compared to spleen preservation ^(10)^. In addition, the JCOG1001 trial evaluated the efficacy of bursectomy (removal of the anterior membrane of the transverse mesocolon and the pancreatic capsule) in patients with cT3 (SS) or cT4a (SE) gastric cancer. The aim was to facilitate complete resection of the regional lymph nodes, along vessels and beneath the peritoneum, and to dissect potential micro peritoneal deposits on the surface of the bursa omentalis. The results showed that bursectomy had no benefit compared to non-bursectomy with respect to survival ^(11)^.

Given these negative results regarding extended surgery, less invasive surgical approaches for preserving gastric function or improving postoperative quality of life have been recently developed, and surgery for gastric cancer has progressed toward standardization and minimally invasive surgery. Less invasive surgery with reduced postoperative complication rates and inflammatory responses should improve long-term outcomes ^(24), (25)^.

Selective lymph node dissection using the sentinel lymph node concept, which plays a pivotal role in breast cancer surgery, has been introduced for early gastric cancer ^(26)^. Radical lymph node dissection may not be necessary in cases where it is known preoperatively that there is no lymph node metastasis. Kitagawa et al. reported sentinel node dissection was safe and effective in early gastric cancer and can be applied despite the complicated nature of gastric lymphatic drainage ^(27)^. Sentinel navigation surgery would facilitate gastric cancer patients to preserve a larger stomach remnant and improve postoperative quality of life. Hence, it is anticipated that sentinel node navigation surgery can be used to achieve highly individualized surgical treatment for patients with early gastric cancer.

## Minimally Invasive Surgery

Major phase III trials comparing laparoscopic surgery with open surgery for gastric cancer are shown in Table 2. Since the JCOG0703 trial ^(28), (29)^, the first phase II trial to conﬁrm the safety of laparoscopy-assisted distal gastrectomy, two phase III trials comparing long-term outcomes after laparoscopic gastrectomy to open gastrectomy for early gastric cancer have been conducted in Japan (JCOG0912) and Korea (KLASS-01) ^(30), (31), (32)^. The non-inferiority of laparoscopic distal gastrectomy (LDG) in patients with non-early gastric cancer has also been investigated in three Asian phase III trials (JLSSG0901, KLASS-02, and CLASS-01) ^(33), (34), (35)^. Most recently, the Chinese CLASS-01 trial demonstrated the non-inferiority of LDG to open distal gastrectomy for patients with non-early gastric cancer ^(35)^.

**Table 2.** Major Phase III Trials Comparing Laparoscopic Surgery with Open Surgery for Gastric Cancer.

Trial name	Published year	No. of patients	Eligible patients	Treatment groups	Morbidity (%)	P-value	Mortality (%)	P-value
JCOG0912 ^(30)^	2017	921	cStage IA/IB	ODG LDG	3.7 3.3	0.72	0 0	-
KLASS-01 ^(31), (32)^	2015, 2016	1416	cStage IA/IB	ODG LDG	19.9 13.0	0.001	0.3 0.6	0.450
JLSSG0901 ^(33)^	2017	507	cT2-T4a	ODG LDG	4.7 3.1	0.285	0.4 0.4	1.000
KLASS-02 ^(34)^	2016	1050	cT2-4a N0-1	ODG LDG	24.3 16.4	0.002	0.6 0.4	0.683
CLASS-01 ^(35)^	2018	1056	cT2-T4a	ODG LDG	15.2 12.9	0.285	0.4 0	0.249

Abbreviations: ODG, open distal gastrectomy; LDG, laparoscopic distal gastrectomy.

Furthermore, robot-assisted gastrectomy (RAG) using the da Vinci Surgical System (Intuitive Surgical, Sunnyvale, CA, USA) is increasingly performed. RAG enables ergonomic surgery by using a flexible robotic arm with multi-joint forceps as well as three-dimensional (3D), high-resolution images; this approach eliminates human tremor, although its drawbacks include high cost and lack of force or tactile sensation. Although several non-randomized, retrospective studies and one single-center, phase II trial suggested that RAG might be feasible for treating early gastric cancer ^(36), (37)^, there have been few prospective multicenter RAG trials, even those focusing only on short-term outcomes. Thus, the safety and efficacy of RAG for patients with non-early gastric cancer remains unclear due to oncological concerns.

## Postoperative Adjuvant Treatment

Postoperative adjuvant treatment is given to prevent relapse due to minimal residual tumor after curative surgical resection. Major phase III trials of adjuvant chemotherapy and chemoradiotherapy for gastric cancer are shown in Table 3. Several trials have shown postoperative adjuvant chemotherapy contributes to improved prognosis in patients who underwent D2 gastrectomy. A small-scale, randomized, controlled trial (NSAS-GC) showed a signiﬁcant survival beneﬁt from postoperative adjuvant chemotherapy with uracil–tegafur (UFT) in patients with T2 N1–2 gastric cancer ^(38)^. The large-scale ACTS-GC trial, which had a dramatic impact on gastric cancer treatment, demonstrated the efficacy of postoperative chemotherapy using S-1 for 12 months after curative D2 gastrectomy in patients with pStage II or III gastric cancer ^(39), (40)^. In addition, the SAMIT trial demonstrated that S-1 was significantly superior to UFT ^(41)^; S-1 monotherapy became the standard treatment for gastric cancer in Japan. The CLASSIC trial also showed the efficacy of postoperative chemotherapy using capecitabine plus oxaliplatin for 6 months after curative D2 gastrectomy in the same target population ^(42)^. Therefore, adjuvant chemotherapy with 12 months of S-1 or 6 months of capecitabine plus oxaliplatin has been a standard treatment after D2 gastrectomy for pStage II or III gastric cancer. In terms of the administration period, the JCOG1104 trial (OPAS-1) compared four courses and eight courses of S-1 adjuvant chemotherapy for pStage II gastric cancer. The JCOG Data and Safety Monitoring Committee recommended early termination of the trial because the point estimate of the hazard ratio (HR) for recurrence was greater than the non-inferiority margin of the HR, a prespecified criterion for early stopping ^(43)^. The study was closed and postoperative S-1 adjuvant chemotherapy for pStage II gastric cancer is continued until one year if feasible. Most recently, the JACCRO GC-07 trial (START-2), comparing 1 year of S-1 alone and 6 cycles of S-1 plus docetaxel followed by S-1 alone until 1 year after surgery in pStage III gastric cancer, showed that survival in the S-1 plus docetaxel group was significantly better than the S-1 alone group at the interim analysis ^(44)^. Based on these results, S-1 alone for 1 year is recommended for patients with pStage II gastric cancer, while S-1 plus docetaxel is recommended for patients with pStage III gastric cancer.

**Table 3.** Major Phase III Trials of Adjuvant Chemotherapy and Chemoradiotherapy for Gastric Cancer.

Trial name	Published year	No. of patients	Eligible patients	Treatment groups	Survival (%)	P-value
ACTS-GC ^(39), (40)^	2007, 2011	1059	pStage II-III (D2 gastrectomy)	no treatment (surgery alone) S-1	3-year OS: 70.1 3-year OS: 80.1	0.003
SAMIT ^(41)^	2014	1495	cT4a-T4b (D2 gastrectomy)	UFT with or without paclitaxel S-1 with or without paclitaxel	3-year DFS: 53.0 3-year DFS: 58.2	0.0048
CLASSIC ^(42)^	2014	1035	pStage II-III (D2 gastrectomy)	no treatment (surgery alone) Capecitabine + oxaliplatin	3-year DFS: 59 3-year DFS: 74	<0.0001
OPAS-1 ^(43)^	2017	528	pStage II	S-1 (6 months) S-1 (12 months)	3-year RFS: 88.9 3-year RFS: 95.3	0.93^*^ (for non-inferiority)
START-2 ^(44)^	2018	951	pStage III	S-1 S-1 + docetaxel	3-year RFS: 49.6 3-year RFS: 65.9	0.0007
INT-0116 ^(45)^	2001	556	pStage IB-III (D0/D1 gastrectomy)	no treatment (surgery alone) 5-FU + LV + RT	3-year OS: 41 3-year-OS: 50	0.005
ARTIST ^(46)^	2015	458	pStage IB-III (D2 gastrectomy)	Capecitabine + cisplatin Capecitabine + cisplatin + CRT (Capecitabine + RT)	3-year DFS: 74.2 3-year DFS: 78.2	0.0862
CRITICS ^(47)^	2018	788	cStage IB-III (D1+/D2 gastrectomy)	ECX or EOX ECX or EOX + RT	5-year OS: 42 5-year OS: 40	0.90

* one-sided p-value. Abbreviations: OS, overall survival; DFS, disease-free survival; RFS, recurrence-free survival; MST, median survival time; UFT, uracil–tegafur; CRT, chemoradiotherapy; RT, radiotherapy; 5-FU, fluorouracil; LV, leucovorin; ECX, epirubicin plus cisplatin plus capecitabine; EOX, epirubicin plus oxaliplatin plus capecitabine.

Regarding chemoradiotherapy, the survival benefit of postoperative chemoradiotherapy after limited D0 or D1 lymph node dissection was demonstrated in the American INT-0116 trial ^(45)^. However, the Korean ARTIST trial showed that this treatment was ineffective after standard D2 lymph node dissection ^(46)^. Furthermore, the recent Dutch CRITICS trial demonstrated no survival benefit from adding radiotherapy to perioperative chemotherapy using epirubicin plus cisplatin or oxaliplatin plus capecitabine after D1+ or D2 lymph node dissection ^(47)^.

## Preoperative Neoadjuvant Treatment

Preoperative neoadjuvant chemotherapy for gastric cancer was initially developed in the West. Major phase III trials are shown in Table 4. In the MAGIC trial, perioperative use of epirubicin, cisplatin, and fluorouracil (ECF) significantly improved survival compared to surgery alone ^(48)^. Based on the trial results, perioperative ECF has long been a standard treatment regimen in the West. Most recently, the FLOT4 trial showed that perioperative chemotherapy with fluorouracil, leucovorin, oxaliplatin, and docetaxel (FLOT) was superior to perioperative ECF or epirubicin, cisplatin, and capecitabine (ECX) for resectable gastric cancer with respect to survival and the proportion of patients who achieved R0 resection ^(49), (50)^. Based on this result, perioperative chemotherapy with FLOT is a new standard therapy for resectable gastric cancer in Western countries. On the other hand, Japan has limited the target population of neoadjuvant treatment to patients with marginally resectable or advanced gastric cancer (the latter defined as macroscopic type 4 or large (≥8 cm) type 3 tumors) and extensive lymph node metastasis (ELM), meaning bulky lymph node metastasis along the celiac artery and its branches and/or para-aortic lymph node metastasis. The JCOG0210 phase II trial showed favorable outcomes for patients with curable macroscopic type 4 or large type 3 gastric cancer ^(51)^, and the JCOG0501 trial evaluated the efficacy of adding neoadjuvant chemotherapy using cisplatin plus S-1 (CS) to D2 gastrectomy followed by adjuvant S-1 chemotherapy. The results did not indicate a survival advantage ^(52)^. Also, another phase II trial (JCOG0405) evaluated two or three cycles of CS followed by D2 plus PAND for patients with ELM. It showed an excellent response rate of 64.7% and a 3-year survival of 58.8% with no treatment-related deaths ^(53)^. Based on the results, preoperative CS chemotherapy is a tentative standard treatment for gastric cancer with ELM in Japan.

**Table 4.** Major Phase III Trials of Neoadjuvant Chemotherapy for Gastric Cancer.

Trial name	Published year	No. of patients	Eligible patients	Treatment groups	R0 resection rate (%)	OS (%)	P-value
MAGIC ^(48)^	2006	503	cStage II-III	Surgery alone ECF (Peri)	66.4 69.3	5-year: 23.0 5-year: 36.3	0.009
FLOT4 ^(49), (50)^	2016, 2017	716	cStage IB-III	ECF/ECX (Peri) FLOT (Peri)	77 84	3-year: 48 3-year: 57	0.012
JCOG0501 ^(52)^	2018	300	Type 3 (≥8cm) or Type 4	CS (Pre) + S-1 (Post) S-1 (Post)	74.2 65.7	3-year: 62.4 3-year: 60.9	0.284^*^

* one-sided p-value. Abbreviations: OS, overall survival; ECF, epirubicin plus cisplatin plus fluorouracil; ECX, epirubicin plus cisplatin plus capecitabine; FLOT, fluorouracil plus leucovorin plus oxaliplatin plus docetaxel; CS, cisplatin plus S-1; Peri, perioperative; Pre, preoperative; Post, postoperative.

A new phase II trial (JCOG1704) of preoperative chemotherapy using docetaxel plus oxaliplatin plus S-1 (DOS) for patients with ELM is now being planned. Furthermore, to evaluate the efficacy of trastuzumab (a monoclonal antibody against human epidermal growth factor receptor type 2 (HER2)) as neoadjuvant chemotherapy, CS plus trastuzumab is being compared to CS alone for patients with HER2-positive ELM in an ongoing, randomized phase II trial (JCOG1301C). The current JCOG1509 trial evaluates the efficacy of neoadjuvant S-1 plus oxaliplatin, followed by adjuvant S-1, compared to adjuvant S-1 alone or S-1 plus docetaxel in cStage IIB–IIIC gastric cancer. In Korea, a phase II trial of neoadjuvant DOS triplet regimen, followed by surgery and adjuvant S-1, in cStage II or III gastric cancer demonstrated promising outcomes, with a 98% R0 resection rate, 90% 2-year disease-free survival, and manageable toxicity ^(54)^. With this background, a subsequent phase III trial (PRODIGY) of neoadjuvant DOS chemotherapy for patients with cStage II or III gastric cancer is now ongoing in Korea.

## First-line Treatment for Unresectable or Recurrent Gastric Cancer

Several retrospective studies suggested that palliative surgery for incurable, advanced, or unresectable gastric cancer contributed to prolonged survival ^(55), (56)^. Against this background, the phase III REGATTA trial compared gastrectomy followed by chemotherapy to chemotherapy alone in terms of survival for advanced gastric cancer patients with a single non-curable factor ^(57)^. However, gastrectomy followed by chemotherapy did not indicate a survival benefit compared to chemotherapy alone. Based on this result, reduction surgery cannot be justiﬁed for treating patients with unresectable gastric cancer, so the standard treatment is chemotherapy.

Major phase III chemotherapy trials for patients with unresectable or recurrent gastric cancer are shown in Table 5. In this population, the JCOG9912 trial demonstrated that S-1 alone was not inferior to fluorouracil with respect to survival ^(58)^. Moreover, the SPIRITS trial demonstrated the survival advantage of CS compared to S-1 alone in these patients ^(59)^. Most recently, the JCOG1013 trial failed to show a survival advantage of a triplet regimen using docetaxel plus CS (DCS) compared to CS ^(60)^. Based on these results, CS has been a standard first-line regimen for patients with unresectable or recurrent gastric cancer in Japan.

**Table 5.** Major Phase III Trials of Chemotherapy for Unresectable or Recurrent Gastric Cancer.

Trial name	Published year	No. of patients	Line of treatment	Treatment groups	Response rate (%)	MST (months)	P-value
JCOG9912 ^(58)^	2009	704	1^st^	5-FU Irinotecan + cisplatin S-1	9 38 28	10.8 12.3 11.4	0.0552^*^ (Irinotecan + cisplatin vs. 5-FU) 0.0005^*^ (for non-inferiority of S-1 vs. 5-FU)
SPIRITS ^(59)^	2008	298	1^st^	S-1 CS	31 54	11.0 13.0	0.04
JCOG1013 ^(60)^	2018	741	1^st^	CS DCS	56.0 59.3	15.3 14.2	0.47^*^
G-SOX ^(61)^	2015	642	1^st^	CS SOX	52.2 55.7	13.1 14.1	0.0583 (for non-inferiority)
REAL-2 ^(62)^	2008	964	1^st^	ECF ECX EOF EOX	40.7 46.4 42.4 47.9	9.9 9.9 9.3 11.2	0.02 (ECF vs. EOX)
ToGA ^(63)^	2010	584	1^st^ (HER2-positive)	Capecitabine/5-FU + cisplatin Capecitabine/5-FU + cisplatin + trastuzumab	35 47	11.1 13.8	0.0046
RAINFALL ^(67)^	2018	645	1^st^	Capecitabine/5-FU + cisplatin Capecitabine/5-FU + cisplatin + ramucirumab	36 41	10.74 11.17	0.68
REGARD ^(65)^	2014	355	2^nd^	Placebo Ramucirumab	3 3	3.8 5.2	0.047
RAINBOW ^(66)^	2014	665	2^nd^	Paclitaxel Paclitaxel + ramucirumab	16 28	7.4 9.6	0.017
ABSOLUTE ^(68)^	2017	741	2^nd^	weekly paclitaxel (w-Ptx) weekly nab-paclitaxel (w-Nab) triweekly nab-paclitaxel (t-Nab)	25 24 33	10.9 11.1 10.3	0.0085^*^ (for non-inferiority of w-Nab vs. w-Ptx) 0.062^*^ (for non-inferiority of t-Nab vs. w-Ptx)
ATTRACTION-2 ^(69)^	2017	493	≥3^rd^	Placebo Nivolumab	0 11.2	4.14 5.26	< 0.0001
KEYNOTE-061 ^(71)^	2018	395	2^nd^	Paclitaxel Pembrolizumab	13.6 15.8	8.3 9.1	0.042^*^

* one-sided p-value. Abbreviations: MST, median survival time; 5-FU, fluorouracil; CS, cisplatin plus S-1; DCS, docetaxel plus cisplatin plus S-1; SOX, S-1 plus oxaliplatin; ECF, epirubicin plus cisplatin plus fluorouracil; ECX, epirubicin plus cisplatin plus capecitabine; EOF, epirubicin plus oxaliplatin plus fluorouracil; EOX, epirubicin plus oxaliplatin plus capecitabine; nab-paclitaxel, nanoparticle albumin-bound paclitaxel.

Oxaliplatin has been considered a key drug instead of cisplatin based on the phase III G-SOX and REAL-2 trial results ^(61), (62)^. Recently, molecular-targeted therapies such as trastuzumab have been introduced for clinical use in patients with advanced gastric cancer. Trastuzumab targets the HER2 receptor, which is overexpressed in up to 20% of gastric cancers. In the ToGA trial, trastuzumab was combined with chemotherapy (capecitabine or fluorouracil plus cisplatin) as a first-line treatment of HER2-positive unresectable or recurrent gastric cancer, and trastuzumab plus chemotherapy was superior to chemotherapy alone in terms of survival ^(63)^. In a Japanese phase II trial (HERBIS-1), trastuzumab plus CS showed a favorable response and survival rate compared to trastuzumab plus chemotherapy in the ToGA trial ^(64)^. Therefore, trastuzumab combined with chemotherapy (capecitabine or S-1 plus cisplatin) is a standard first-line treatment for patients with HER2-positive unresectable or recurrent gastric cancer.

## Second or Third-line Treatment for Unresectable or Recurrent Gastric Cancer

Ramucirumab is a monoclonal antibody that targets VEGFR2, preventing ligand binding and receptor-mediated pathway activation in endothelial cells. In the REGARD trial, ramucirumab prolonged survival compared to placebo in patients with metastatic gastric cancer that had progressed after first-line chemotherapy ^(65)^. In addition, the RAINBOW trial demonstrated that combining ramucirumab with paclitaxel signiﬁcantly improved survival compared to placebo plus paclitaxel in patients with previously treated gastric cancer ^(66)^. Based on these results, the combination of ramucirumab and paclitaxel is a standard second-line treatment for patients with unresectable or recurrent gastric cancer. However, adding ramucirumab to capecitabine or fluorouracil plus cisplatin as a first-line treatment failed to prolong overall survival in the RAINFALL trial ^(67)^. Additionally, the ABSOLUTE trial showed that weekly nanoparticle albumin-bound paclitaxel (nab-paclitaxel) was not inferior to weekly, solvent-based paclitaxel in terms of survival and could be a useful second-line treatment for patients with unresectable or recurrent gastric cancer ^(68)^.

Immunotherapy is attracting significant attention in various cancers. The ATTRACTION-2 trial demonstrated the survival advantage of nivolumab, a monoclonal antibody inhibitor of programmed death-1 (PD-1), compared to placebo for patients with unresectable or recurrent gastric cancer who had been previously treated with two or more chemotherapy regimens ^(69)^. Based on the results, nivolumab monotherapy is a standard third-line treatment for patients with unresectable or recurrent gastric cancer.

Like nivolumab, pembrolizumab is an immune checkpoint inhibitor and a selective monoclonal antibody designed to bind to PD-1; in the KEYNOTE-059 trial, it showed a manageable toxicity proﬁle and promising anti-tumor activity in patients with previously treated metastatic gastric cancer ^(70)^. However, the subsequent phase III trial (KEYNOTE-061) could not demonstrate a survival advantage of pembrolizumab compared to paclitaxel in patients with previously treated metastatic PD-L1–positive gastric cancer ^(71)^. It is necessary to investigate new, effective biomarkers that can predict the effect of these immune checkpoint inhibitors.

## Conclusion

Extended surgery beyond standard D2 gastrectomy does not increase survival in any phase III trials. Minimally invasive surgical approaches such as laparoscopic, robot-assisted, and sentinel node navigation surgery for early gastric cancer have been developed in recent years. Various chemotherapy regimens have shown survival benefits in both perioperative and metastatic settings. In addition, molecular-targeted therapies have become popular for treating unresectable or recurrent gastric cancer. Novel immune checkpoint inhibitors have also demonstrated efficacy in terms of survival for patients with metastatic gastric cancer. Currently ongoing clinical trials are expected to yield further individualized treatments for gastric cancer in the future.

## Article Information

### Conflicts of Interest

Yukinori Kurokawa received grant supports from Taiho Pharmaceutical; Masaki Mori received grant supports from Taiho Pharmaceutical, Novartis Pharma, Chugai Pharmaceutical, Eli Lilly, Daiichi Sankyo, Yakult Honsha, Ono Pharmaceutical, Tsumura, Takeda Pharmaceutical, Asahi Kasei Pharma, Kaken Pharmaceutical, and Pfizer Japan; Yuichiro Doki received grant supports from Taiho Pharmaceutical, Chugai Pharmaceutical, Daiichi Sankyo, Yakult Honsha, Shionogi, Nestle, Astellas Pharma, Tsumura, EA Pharma, Nippon Kayaku, Kaken Pharmaceutical, and Pfizer Japan.

### Author Contributions

Tadayoshi Hashimoto and Yukinori Kurokawa contributed equally to this work.
